# Mitochondrion-Directed Nanoparticles Loaded with a Natural Compound and a microRNA for Promoting Cancer Cell Death via the Modulation of Tumor Metabolism and Mitochondrial Dynamics

**DOI:** 10.3390/pharmaceutics12080756

**Published:** 2020-08-11

**Authors:** Yu-Li Lo, Chen-Shen Wang, Yen-Chun Chen, Tse-Yuan Wang, Yih-Hsin Chang, Chun-Jung Chen, Ching-Ping Yang

**Affiliations:** 1 Institute of Pharmacology, National Yang-Ming University, Taipei 112, Taiwan; peach50505@gmail.com (C.-S.W.); mikechen023@gmail.com (Y.-C.C.); alanwang.twn@gmail.com (T.-Y.W.); 2 Center for Advanced Pharmaceutics and Drug Delivery Research, National Yang-Ming University, Taipei 112, Taiwan; 3 Department of Biotechnology and Laboratory Science in Medicine, National Yang-Ming University, Taipei 112, Taiwan; cyh@ym.edu.tw; 4 Department of Medical Research, Taichung Veterans General Hospital, Taichung City 407, Taiwan; cjchen@vghtc.gov.tw (C.-J.C.); milkygp@gmail.com (C.-P.Y.)

**Keywords:** EGFR targeting, mitochondrion-directed nanoparticles, natural compound, microRNA, mitochondrial dynamics, cancer

## Abstract

Mitochondrial dysfunction may cause cancer and metabolic syndrome. Ellagic acid (abbreviated as E), a phytochemical, possesses anticancer activity. MicroRNA 125 (miR-125) may regulate metabolism. However, E has low aqueous solubility, and miR-125 is unstable in a biological fluid. Hence, this study aimed to develop nanoparticle formulations for the co-treatment of miR-125 and E. These nanoparticles were modified with one mitochondrion-directed peptide and a tumor-targeted ligand, and their modulating effects on mitochondrial dysfunction, antitumor efficacy, and safety in head and neck cancer (HNC) were evaluated. Results revealed that miR-125- and E-loaded nanoparticles effectively targeted cancer cells and intracellular mitochondria. The co-treatment significantly altered cellular bioenergetics, lipid, and glucose metabolism in human tongue squamous carcinoma SAS cells. This combination therapy also regulated protein expression associated with bioenergenesis and mitochondrial dynamics. These formulations also modulated multiple pathways of tumor metabolism, apoptosis, resistance, and metastasis in SAS cells. In vivo mouse experiments showed that the combined treatment of miR-125 and E nanoparticles exhibited significant hypoglycemic and hypolipidemic effects. The combinatorial therapy of E and miR-125 nanoparticles effectively reduced SAS tumor growth. To our best knowledge, this prospective study provided a basis for combining miRNA with a natural compound in nanoformulations to regulate mitochondrial dysfunction and energy metabolism associated with cancer.

## 1. Introduction

Epithelial–mesenchymal transition (EMT) and chemoresistance are two major challenges of the successful treatment of various tumors, such as oral squamous cell carcinoma (OSCC), a type of head and neck cancer (HNC) [1]. Alterations in mitochondrial dynamics and mitophagy are associated with the chemoresistance and EMT of tumor cells [2]. Mitochondrial dynamics is misbalanced when the mitochondria change from a highly organized and elongated network (mitochondrial fusion) into a fragmented and punctate form (fission) [3]. Furthermore, the suppression of mitochondrial fission is involved in cellular survival, neoplastic progression, and drug resistance [4].

Mitophagy may act as a pro-survival method of cells under inflammation by maintaining the mitochondrial dynamic balance. Mitophagy has become a potential anticancer target by triggering mitochondrial dysfunction in cancer cells because of the imperative role of mitophagy on mitochondrial homeostasis and cell survival. Remarkably, phosphatase and tensin homolog (PTEN)-induced putative kinase 1 (PINK1)/Parkin-related mitophagy is usually activated by mitochondrial damage [5]. Dysfunctional mitochondria with reduced mitochondrial membrane potential (MMP) may activate PINK1 and then trigger Parkin to be overexpressed on the surfaces of the mitochondria, thereby inducing mitophagy [6]. PINK1/Parkin- dependent mitophagy plays an important role in mitochondrial homeostasis [7], and the modulation of PINK1/Parkin-mediated mitophagy alters the sensitivity of multidrug-resistant cancer cells to anticancer agents [8]. Accumulating evidence has indicated that the inhibition of Parkin-related mitophagy increases cancer cell death [9]. Recently, tanshinone IIA, a potential antitumor agent, has been found to suppress the adenosine monophosphate-activated protein kinase (AMPK) pathway and inactivate Parkin, thus decreasing mitophagy activity and enhancing colorectal cancer (CRC) cell apoptosis [10]. A similar series of events occurs in various tumor types [6,11]. Nevertheless, contradictory findings have suggested that the activation of mitophagy may improve the cytotoxicity of drugs against various cancers [12]. For example, zinc oxide nanoparticles increase the intracellular levels of reactive oxygen species (ROS) and reduce the MMP, but these nanoparticles stimulate the PINK1/Parkin-mediated mitophagy to induce the anticancer activity in tongue cancer CAL 27 cells [13]. Thus, the role of mitophagy in cancer progression or inhibition should be clarified.

Ellagic acid (abbreviated as E), a natural polyphenolic phytochemical, can be isolated from edible plants, algae, and mushroom [14]. Recent studies have shown that E possesses anticancer, anti-inflammatory, and antioxidative activities [15]. Consistently, E may reduce metabolic syndromes caused by high-fat diets (HFDs) in rats [16]. Furthermore, a natural product extract containing E increases the sensitivity of CRC cells to 5-fluorouracil (5-FU) therapy by triggering a ROS-mediated and mitochondrion-associated apoptotic pathway via the induction of mitochondrial dysfunction and autophagy [17].

MicroRNA 125 (miR-125), a miRNA of 22 nucleotides located at chromosome 19q13, is found to have commonly lower expression in several tumor types, including CRC, HNC, ovarian cancer, lung cancer, and medulloblastoma [18,19,20]. miR-125 suppresses CRC cell growth by targeting and decreasing the expression of vascular endothelial growth factor, triggering CRC cell apoptosis [19]. Interestingly, oncogenic long noncoding RNA may regulate glycolysis by binding to miR-125 and affect the progression of esophageal squamous cell carcinoma cells [20]. miR-125 modulates the gene expression levels in lipid and glucose utilization [21]. Additionally, miR-125 may affect oxygen consumption and mitochondrial DNA content, modulating mitochondrial bioenergenesis and respiration [21]. These studies have suggested the critical role of miR-125 in regulating lipid and glucose metabolism, mitochondriogenesis, and cancer cell apoptosis.

However, miRNA is unstable in biological systems because of its rapid degradation by endonucleases and exonucleases and fast elimination from the body. Furthermore, it experiences difficulty in penetrating the cell membrane because of its negative charge [22]. Hence, we proposed cationic low-toxicity solid lipid nanoparticles (SLN) to prevent miR from degradation and clearance and reach intracellular target sites. SLN, consisting of a cationic lipid and a surfactant, may form a stable mixture with miR-125 via charge attraction. SLN also possesses the characteristics of physical stability and protective property of miRNA [22]. Therefore, we prepared miR-125 in SLN to function as a combination therapeutic agent to enhance the effect of E as an effective antineoplastic agent by regulating mitochondrial dynamics in poorly differentiated human squamous SAS cell carcinoma of the tongue (one OSCC cell line).

Furthermore, E has poor aqueous solubility, rapid body clearance, and low bioavailability. In addition, its clinical application is limited by its fast metabolism and enzymatic degradation [23]. Thus, we designed lipid-polymer nanoparticles (LPNs) to improve the aforementioned problems of E. LPNs are lipid-based delivery systems of drugs or natural compounds. LPNs are usually composed of a surface phospholipid shell and a biocompatible polymer core, such as poly(lactic-co-glycolic acid; PLGA), an FDA-approved biodegradable polymer [24]. This structure combines the advantages of a lipophilic compound/PLGA nanocore and a lipid shell [25].

Thus, the design of LPN and SLN in the present study may prevent the metabolism and degradation of E and miR-125 and increase their systemic stability to improve the cellular uptake of the encapsulated cargos. Further coupling of LPN and SLN with peptides/ligands of different functions can provide a tumor-specific targeting delivery [26]. Hence, we designed nanoparticles by modifying their surfaces with two functional peptides that targeted cancer cells to improve cellular penetration and subcellular mitochondrial specificity. The targeting of LPN and SLN to specific receptors overexpressed in tumor cells may reduce the toxicity to normal cells [24]. Additionally, the mitochondrion-directed nanoparticles may display the potential to modulate mitochondria-mediated cancer cell death [27].

L peptide is screened from the phage display peptide library with good epidermal growth factor receptor (EGFR) binding and tumor targeting characteristics [28]. This peptide also possesses a prototypic CendR motif responsible for triggering extravasation and tumor penetration [28]. K peptide is screened with remarkable mitochondrion and tumor-focused characteristics. Previous studies have demonstrated that K peptide has a mitochondrial membrane disrupting function and can enhance the intracellular accumulation of nanoparticles via the electrostatic interaction between a lysine group and an anionic tumor cell surface [29,30]. The cationic domain of this peptide disrupts the negatively charged mitochondrial membranes, which have a high transmembrane potential [31]. In our study, K peptide was verified in terms of its mitochondrial targeting property and penetrating effect on cancer cells. Hence, the lipid shell of SLN and LPN was modified with L peptide to target EGFR-overexpressing SAS tumors and with M peptide to direct the mitochondria and enhance cancer penetration for SLN-KL and LPN-KL formulations. Furthermore, miR-125 and E were incorporated into SLN-KL and LPN-KL to prepare miR-125/SLN-KL and E/LPN-KL, respectively. Thus, due to the potential roles of miR-125 and E in regulating mitochondrial dynamics, apoptosis, and migration of cancer cells, we aimed to explore the effects and mechanisms of the combinatorial therapy of miR-125/SLN-KL and E/LPN-KL in suppressing HNC via multiple signaling modulations of tumor energy metabolism, metastasis, and resistance in vitro and in vivo.

## 2. Materials and Methods

### 2.1. Materials

E, lecithin, distearoylphosphatidylethanolamine (DSPE)-polyethyleneglycol molecular weight 2000 (PEG2000), and PLGA were purchased from Sigma-Aldrich (St. Louis, MO, USA). miR-125 and FAM-miR125 were purchased from GenePharma (Shanghai, China). Peptide K and L were bought from Kelowna Biotech (Taipei, Taiwan) at >95% purity. Monoclonal antibodies (Mab) of B-cell lymphoma 2 (Bcl-2), Bcl-2 associated X-protein (Bax), *β*-catenin, caspase 9, caspase 3, poly (ADP-ribose) polymerase (PARP), receptor-interacting protein (RIP)1, RIP3, Slug, E-cadherin, etc. were bought from Cell Signaling Technology (Beverly, MA, USA), GeneTex (Irvine, CA, USA), Jackson (West Grove, PA, USA), or Abcam (Cambridge, UK), as demonstrated in Supplementary Table S1. All cell culture medium and reagents were obtained from Promega (Madison, WI, USA), Invitrogen (Carlsbad, CA, USA), Gibco BRL (Grand Island, NY, USA), or Hyclone (Logan, UT, USA). All other chemical reagents were purchased from either Merck (Darmstadt, Germany) or Sigma-Aldrich.

### 2.2. Synthesis and Preparation of E/LPN-KL and miR125/SLN-KL

DSPE-PEG was mixed with peptide K or L at a molar ratio 1:1 for 24 h at room temperature to synthesize K or L-linked lipids. The products were put in a dialysis bag against water. The purified K or L-bound lipids were freeze-dried and detected by matrix-assisted laser desorption ionization time-of-flight mass spectrometry (MALDI-TOF MS; Bruker, Bremen, Germany).

E/LPN-KL was prepared by an o/w emulsion method by dispersing 3 mg/mL of mPEG-DSPE or peptide-conjugated lipid and lecithin in ethanol/water (1:1) solution. E in PLGA (50:50 LA:GA) was prepared at a concentration of 2 mg/mL and added to the above dispersion dropwise. The resulting dispersion was mixed by sonication for 10 min. The resulting E-loaded nanoparticles were stirred using a centrifugal evaporator to allow the solvent to vaporize completely and then stored at 4 °C.

SLN was prepared by mixing lecithin, 1, 2-dioleoyl-3-trimethylammonium-propane (DOTAP), DSPE-PEG-peptide at a molar ratio of 1:0.1:0.1 in methanol/dichloromethane (2:1). The dispersion was added into Tween 80 solution drop by drop. A miR-125 solution of 100 nM was incorporated into SLN-KL and incubated at room temperature for 30 min to form a final dispersion of miR125/SLN-KL.

### 2.3. Size Distribution and Zeta Potential of LPN and SLN

The size distribution and zeta potential of LPN and SLN were detected using a Zetasizer nano ZS analyzer (Malvern Instruments Ltd., Malvern, Worcestershire, UK). Data were computed by a Malvern software to acquire the values of the polydispersity index.

### 2.4. The Shape and Particle Morphology by Transmission Electron Microscopy (TEM)

The surface morphology of nanoparticles was observed using a transmission electron microscopy (TEM) system (JEM-2000EXII, Tokyo, Japan). One drop of the sample solution was mounted onto a carbon-coated copper grid and then visualized under TEM.

### 2.5. Encapsulation Efficiency (EE%)

Unbound E or miR was separated from the loaded LPN or SLN by centrifugation. E or miR in the filtrate was analyzed by HPLC or NanoDrop (Thermo Fisher, MA, USA). The HPLC system composed of a pump (L7100; Hitachi, Tokyo, Japan), accompanied with an automated injector (L2200), a 5 μm LiChrospher column (25 cm long; Merck, Darmstadt, Germany), and an ultraviolet detector (L2400; Hitachi, Tokyo, Japan). The mobile phase included 60% methanol and water (gradient solvent from 50:50 to 70:30, *v/v*) plus o-phosphoric acid and ran at a flow rate of 1 mL/min. The solvent/water was pre-degassed using a sonicator. The detection wavelength was set at 259 nm.

EE% was calculated as the percentage of the amount of E or miR in LPN or SLN divided by the total amount of added E or miR. EE% and DL% were calculated by Equation (1), as shown below.

EE% = [(W_e_ − W_f_)/W_e_] × 100% (1)

where W_e_ is the weight of added E or miR, and W_f_ is the weight of E or miR in the filtrate.

### 2.6. Cell Lines

SAS and normal oral keratinocyte (NOK) cells, obtained from the lab of Professor Muh-Hwa Yang (National Yang-Ming University), were incubated in DMEM containing 10% FBS and 1% penicillin/streptomycin at 37 °C in an incubator of 5% CO_2_ and 90% relative humidity. All the cell culture reagents were purchased from Gibco (Grand Island, NY, USA) or HyClone (Logan, UT, USA).

### 2.7. Identification of Intracellular Localization

DiI (1,1′-Dioctadecyl-3,3,3′,3′-tetramethylindocarbocyanine perchlorate; Thermo Fisher, Waltham, MA, USA) was used as a lipophilic fluorescent probe of E, and miR-125 was labeled with FAM (carboxyl fluorescein; GenePharma). SAS cells were seeded overnight and incubated with DiI//LPN-KL or FAM-miR/SLN-KL for the indicated time. These cells were then fixed with 4% paraformaldehyde for 10 min and stained with 4′,6-diamidino-2-phenylindole (DAPI; blue) at 37 °C to identify the nucleus. For DiI//LPN-KL intracellular localization, MitoTracker^®^ Green (MitoGreen), LysoTracker^®^ Green (LysoGreen), and an antibody against EGFR were added to monitor mitochondrial, lysosomal, and EGFR distribution. For the intracellular distribution of FAM-miR/SLN-KL (green), MitoTracker^®^ Red (MitoRed; Thermo Fisher), LysoTracker^®^ Red (LysoRed; Thermo Fisher), and an antibody against EGFR were used to detect mitochondrial, lysosomal, and EGFR localization. For both nanoparticle formulations, early endosomes were identified by immunofluorescence staining with an antibody against early endosome antigen 1 (EEA1) overnight. Images were obtained using a confocal laser scanning microscope (CLSM; Olympus FV10i; Olympus, Tokyo, Japan) with a 60× objective lens at a magnification of 1500×. The representative images are shown (*n* = 3).

### 2.8. Detection of Mitochondrial ROS Level Using Mito-SOX

MitoSOX (Thermo Fisher) was used to monitor mitochondrial reactive oxygen species (ROS), especially superoxide. After treatment, 5 × 10^5^ cells were mixed with 5 μM of MitoSOX reagent at 37 °C in the dark for 20 min. After harvest, the fluorescence intensity of MitoSOX was detected using a FACSCalibur flow cytometer (BD Biosciences, San Jose, CA, USA) with excitation at 510 nm and emission at 580 nm [32]. The percentage change of mitochondrial ROS level by the treatment group is expressed relative to that of the control (CTR) without treatment (only medium) group.

### 2.9. Measurement of Total Cellular ATP

After treatment, the ATPlite luminescence One-Step ATP detection assay (Perkin Elmer, Waltham, MA, USA) was used to monitor ATP levels, based on firefly luciferase/luciferin activity measurement at 560 nm using a TECAN Infinite 200 multimode microplate reader (Tecan Inc., Mannedorf, Switzerland) [33].

### 2.10. Measurements of Mitochondrial Respiration

Extracellular acidification rate (ECAR) and the oxygen consumption rate (OCR) were evaluated using the Seahorse XF Glycolysis Stress Test Kit and Seahorse XF Cell Mito Stress Test Kit (Agilent Technologies, Palo Alto, CA, USA), respectively. After pretreatment with oligomycin (ATP synthase inhibitor; 1 μM), different formulations of E and/or miR-125-loaded formulations were treated, followed by injection of carbonylcyanide p-trifluoromethoxyphenylhydrazone (FCCP; protonophoric uncoupler; 1 μM) and antimycin A (electron transport chain inhibitor; 1 μM). OCR and ECAR were detected using a Seahorse XFe24 Extracellular Flux Analyzer (Seahorse Biosciences, North Billerica, MA, USA).

### 2.11. Glucose Uptake Assay

After treatment for the indicated time, cells were incubated with fluorescent D-glucose analog (2-[N-(7-nitrobenz-2-oxa-1,3-diazol-4-yl) amino]-2-deoxyglucose, 2-NBDG) for 10 min in the dark, washed by PBS, and then added with cell lysis buffer to prepare the cell extracts. These cell extracts were homogenized and centrifuged. The supernatants were transferred into 96-well, and the fluorescence was measured using a TECAN reader with excitation and emission at 485 and 535 nm, respectively.

### 2.12. Oil Red O Staining for Intracellular Lipid Accumulation

After treatment, the cells were stained with Oil Red O (Sigma-Aldrich). The cells were washed twice with PBS, fixed with paraformaldehyde in PBS at room temperature for 1 h. The cells were then washed with deionized water, stained with Oil Red O for 1 h in the dark. The stained lipid droplets were determined by measuring the absorbance at 492 nm using a TECAN reader. The percentage change of lipid accumulation by the treatment group is expressed relative to that of the CTR group [34].

### 2.13. Evaluation of Protein Expression Levels via Western Blot

After treatment, cells were lysed in an ice-cold lysis solution containing proteinase inhibitor to reduce protein degradation. The protein concentrations of supernatants were measured by BCA protein assay. Protein samples were run by SDS-polyacrylamide gel electrophoresis and then transferred onto polyvinylidene difluoride membranes. The blots were blocked in 5% nonfat milk for 1 h and then incubated at 4 °C with primary antibodies against different proteins from Cell Signaling or Abcam (Supplementary Table S1). The samples were then added with horseradish peroxidase-conjugated secondary IgG. These blots were developed by enhanced chemiluminescence kits (PerkinElmer) and visualized with a Millipore detection system.

### 2.14. Migration Assay

After overnight seeding of SAS cells in inserts (Ibidi GmbH, Munich, Germany), a sterile pipette tip was used to make a scratch in each well of cancer cells. The cells were then treated with the different formulations and monitored for 15 h. Images were photographed at the same site under a light microscope, and the migration area was calculated using Image J. Relative migration area% was computed according to the following equation:

Relative migration area (% of area at 0 h) = 100% − [control area (15 h)/control area (0 h) × 100%](2)

### 2.15. Cytotoxicity by Sulforhodamine B (SRB) Assay

After overnight seeding of cells, various formulations of E with or without miR-125/SLN-KL were added to SAS or NOK cells incubated in 96-well plates at 37 °C for 48 h. The cytotoxic effects were determined by SRB assay. The cells were stained with 0.04% SRB for 10 min and washed with 1% acetic acid. Finally, the absorbance at 540 nm was monitored using a TECAN reader.

### 2.16. Apoptosis Detection Assay

After treatment, staining on the cells was performed with Annexin V-FITC/propidium iodide (PI) laboring solution for 15 min in the dark. Cell population % in apoptosis and necrosis was detected and analyzed by a BD flow cytometer.

### 2.17. Establishment of In Vivo SAS-Tumor Bearing Mouse Model

BALB/c nude mice (6 weeks old, ~22 g body weight) were obtained from the National Laboratory Animal Center and maintained in the Animal Center of National Yang-Ming University. All mice were kept on a half-day light-dark cycle. Animal care and handling procedures obey the guidelines and have been approved by the Institutional Animal Care and Use Committee. SAS cells were administered subcutaneously into the right cheek region of the male mice. Tumor size was calculated according to the following equation:

V = (L × W^2^)/2 (3)

where L (length, mm) is the longest diameter, and W (width, mm) is the shortest diameter perpendicular to the longest axis.

### 2.18. Evaluation of Antitumor Efficacy on SAS Tumor-Bearing Mice

The tumor size of mice was grown up to about 60 mm^3^ before treatment. SAS tumor-bearing mice were randomly divided into five groups (*n* = 5), namely, saline (control; CTR), E (80 mg/kg), E/LPN, E/LPN-KL, and E/LPN-KL +miR125/SLN-KL (E, 80 mg/kg; miR-125, 1.25 mg/kg). Each group received different formulations every 2 days for 20 days. The tumor size of the mice was measured with a digital caliper every 2 days, and the tumor volume (V) was computed according to Equation (3).

### 2.19. Biochemical Tests

Two days after the final treatment, the whole blood of 150 μL was taken from the orbital sinus of mice. These blood samples were centrifuged to isolate the serum. The serum concentrations of glucose, cholesterol, glutamate pyruvate transaminase (GPT), creatinine (CRE), and creatine kinase-myocardial band (CKMB) were measured using a dry chemistry analyzer (FUJI DRI-CHEM 7000V, FUJIFILM Corporation, Tokyo, Japan) [35].

### 2.20. Statistical Analysis

Data are presented as the means ± standard deviation. Student’s *t*-test was used to analyze differences between the two treatment groups. A significant difference was set at *p* < 0.05.

## 3. Results

### 3.1. Physicochemical Characteristics of E- or miR-125-Loaded Nanoparticles

**Figure 1:**
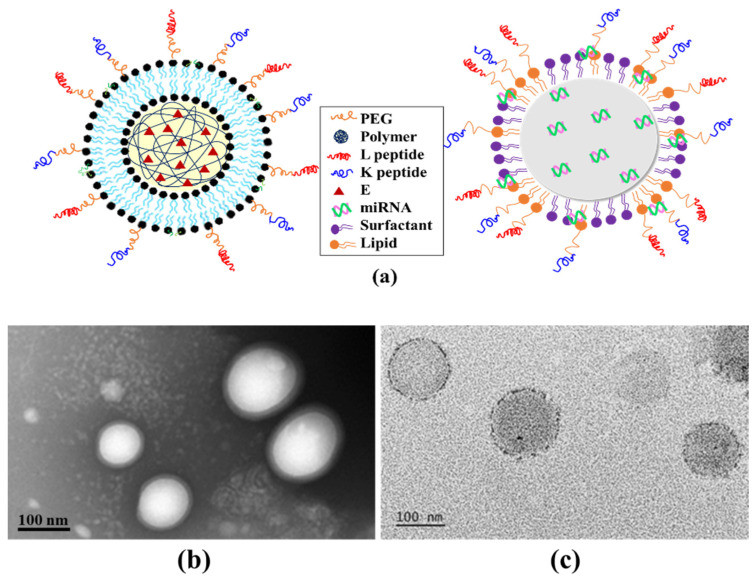

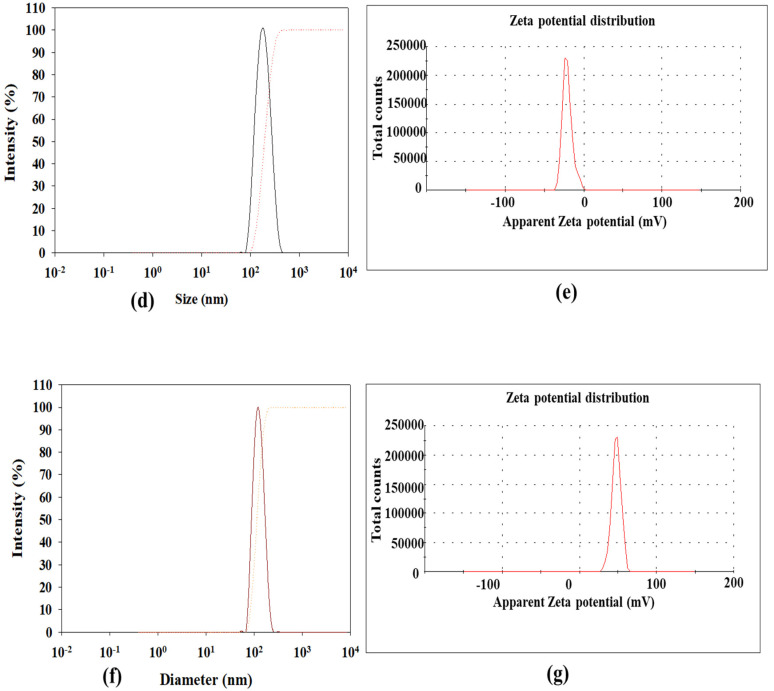
Schematic, morphological characteristics, particle size, and zeta potential of E/LPN-KL and miR-125/SLN-KL formulations. (**a**) A schematic of E/LPN-KL and miR-125/SLN-KL, (**b**,**c**) TEM images of (**b**) E/LPN-KL and (**c**) miR-125/SLN-KL, (**d**) size distribution and (**e**) zeta potential of E/LPN-KL, and (**f**) size distribution and (**g**) zeta potential of miR-125/SLN-KL. The representative plots are shown (*n* = 3). E, ellagic acid; LPN, lipid-polymer nanoparticle; SLN, solid lipid nanoparticles.

The physicochemical characteristics of E/LPN-KL and miR-125/SLN-KL are shown in Figure 1. The size, zeta potential, polydispersity index (PdI), and encapsulation efficiency (EE%) of these E- or miR-125-loaded nanoparticles are presented in Table 1. The schematic of miR125/SLN-KL or E/LPN-KL is displayed in Figure 1a. E/LPN-KL was spherical and had a homogeneous polymer core and a lipid shell modified with peptide K and L (Figure 1b). Similarly, miR125/SLN-KL also displayed spherical shape and had a homogeneous matrix and a surfactant/lipid shell coated with peptide K and L (Figure 1c).

The size, zeta potential, PdI, and encapsulation efficiency (EE%) of these E- or miR-125-loaded nanoparticles are demonstrated in Figure 1d–g and Table 1. The zeta potential of E/LPN-KL was around −19 mV, indicating a negative charge due to the PLGA core (*n* = 3; Table 1, Figure 1e). However, miR125/SLN-KL possessed a positive zeta potential of about 46 mV (*n* = 3; Table 1, Figure 1g), primarily because of the positive charges of cationic lipids and peptides K and L. Interestingly, miR-125 might also be distributed on the surface of the nanoshell, possibly because of the electrostatic interaction between anionic miR and cationic lipids/peptides (Figure 1a–g).

### 3.2. Cellular Internalization of DiI/LPN-KL and FAM-miR125/SLN-KL into SAS Cells

**Figure 2:**
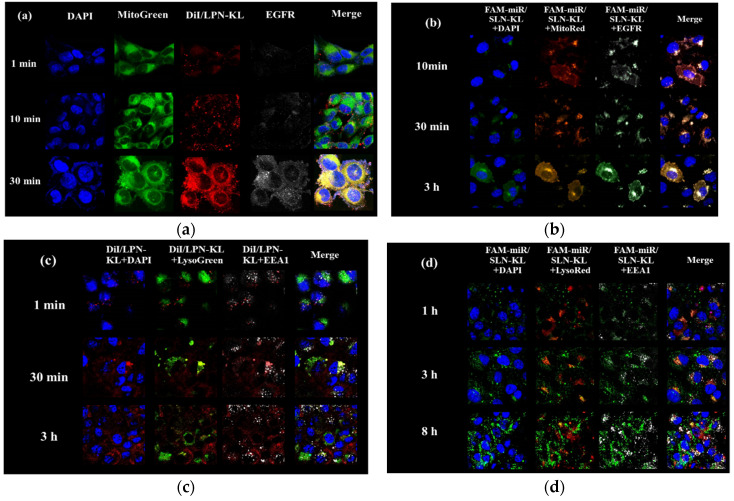
Cellular internalization and uptake of DiI or FAM-miR125/SLN-KL into human tongue squamous carcinoma SAS cells. (**a**,**b**) SAS cells were treated with (**a**–**c**) DiI in LPN-KL or (**b**–**d**) FAM-miR125/SLN-KL for the indicated time intervals and observed with CLSM. (**a**) Green, MitoGreen; red, DiI; blue, DAPI; gray, EGFR. (**b**) Green, FAM-miR125; red, MitoRed; blue, DAPI; gray, EGFR. (**c**) Green, LysoGreen; red, DiI; blue, DAPI; gray, EEA1. (**d**) Green, FAM-miR125; red, LysoRed; blue, DAPI; gray, EEA1. Magnification: 1500×. The representative images are shown (*n* = 3). DiI, 1,1′-Dioctadecyl-3,3,3′,3′-tetramethylindocarbocyanine perchlorate; FAM, carboxyl fluorescein; EEA1, early endosome antigen 1.

The intracellular localization of DiI/LPN-KL and FAM-miR125/SLN-KL in SAS cells was detected via CLSM, as shown in Figure 2. After treatment of DiI/LPN-KL (the molar ratio of DiI: lipid = 1: 10) for the indicated time, the cells were stained with DAPI (blue), MitoTracker Green (MitoGreen; green), and anti-EGFR antibody (gray) to localize the nucleus, mitochondria, and EGFR, respectively (Figure 2a). The intracellular localization of DiI/LPN-KL in SAS cells was visualized after 1, 10, and 30 min of incubation. After these three treatment periods, red fluorescence of DiI appeared mainly in the cytoplasm and mitochondria (Figure 2a). Mitochondrial targeting of DiI was the most predominant after 30 min of incubation (Figure 2a; bottom panels). A similar cellular distribution was also observed in FAM-miR/SLN-KL (Figure 2b). After the treatment of FAM-miR125 (100 nM)/SLN-KL for the specified time, the cells were stained with DAPI (blue), MitoRed (red), and anti-EGFR antibody (gray) to indicate the nucleus, mitochondria, and EGFR, respectively (Figure 2b). EGFR targeting of green FAM-miR/SLN-KL was observed after 10 and 30 min. However, the cytoplasmic and mitochondrial localizations of FAM-miR were noticeable after 3 h of incubation (Figure 2b; bottom panels).

Endosomal escape is critical for the efficacious transport of nanoparticles to subcellular targets [36]. The cellular uptake of DiI/LPN-KL (red) was detected after 1 min, 30 min, and 3 h of incubation (Figure 2c). The cells were then stained with DAPI (blue), LysoGreen (green), and anti-EEA1 antibody (gray) to localize the nucleus, lysosome, and endosome, respectively. Few red dots of DiI were observed in the endosomes at 1 min (Figure 2c; up panels), and the co-localization with lysosomes (yellow) was detected at 30 min (Figure 2c; middle panels). Furthermore, the red fluorescence of DiI was not co-localized with the green fluorescence of lysosomes, indicating that DiI was transported into the cytoplasm after delivery in SAS cells for 3 h (Figure 2c; bottom panels). A similar cellular distribution was also found in FAM-miR/SLN-KL. Partial endosomal and lysosomal entrapment of green FAM-miR was detected after 1 and 3 h (Figure 2d; up and middle panels). However, the cytoplasmic localization of FAM-miR was obvious after 8 h of incubation (Figure 2d; bottom panels).

### 3.3. Effect of Different Treatments on Mitochondrial ROS Production and Bioenergenesis in SAS Cells

**Figure 3:**
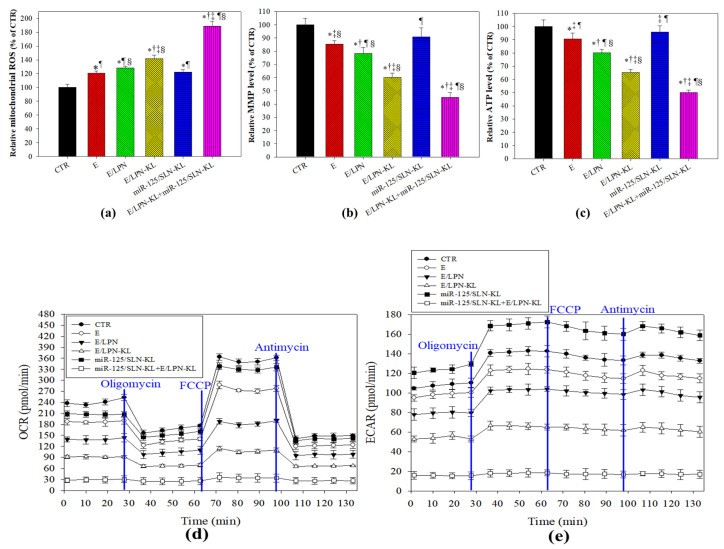
Effect of different treatments on mitochondrial reactive oxygen species (ROS), bioenergenesis, and dynamics in SAS cells. (**a**) Relative ROS percentage. (**b**) Relative ATP percentage. (**c**) Relative mitochondrial membrane potential (MMP) percentage. (**a**–**c**) Control (CTR) was normalized as 100%. The mean fluorescence intensity of the other treatments was normalized relative to CTR. Data are presented as means ± SD from *n* = 3. * *p* < 0.05 compared with CTR, ^†^ *p* < 0.05 compared with E, ^‡^ *p* < 0.05 compared with E/LPN, ^¶^ *p* < 0.05 compared with E/LPN-KL, and ^§^ *p* < 0.05 compared with miR-125/SLN-KL. (d) Oxygen consumption rate (OCR) and (e) extracellular acidification rate (ECAR) related to mitochondrial dynamics in SAS cells.

The imbalance in mitochondrial oxidative metabolism may cause an increase in ROS levels, subsequently inducing oxidative stress and ultimately triggering the disruption in mitochondrial membrane potential (MMP; ΔΨ_m_) and apoptosis in various cancer cells [37,38]. In our study, after the SAS cells were treated with E, E/LPN, or E/LPN-KL, the relative mitochondrial ROS percentages, as indicated by the Mito-SOX fluorescence, were significantly higher than that of the control (Figure 3a). This mitochondrial ROS% was the highest among all the formulations after the cells were incubated with E/LPN-KL+miR-125/SLN-KL (*p* < 0.05; Figure 3a). By contrast, ΔΨ_m_ or ATP levels after the treatment with E, E/LPN, or E/LPN-KL were considerably lower than those of the control (Figure 3b,c). Furthermore, E/LPN-KL plus miR-125/SLN-KL generated the highest degree of decrease in ΔΨ_m_ or ATP levels among all the formulations (*p* < 0.05; Figure 3b,c).

Moreover, oligomycin (the ATP synthase inhibitor), FCCP (uncoupler), and antimycin (electron transport chain inhibitor) were used to evaluate mitochondrial function [39]. We found that the co-treatment of E/LPN-KL and miR125/SLN-KL diminished mitochondrial respiration and aerobic glycolysis to reduce the energy supply in SAS cells, as measured by a seahorse bioenergetics analyzer (Figure 3d,e). Previous works have emphasized that the change in cellular metabolic activities may modulate tumor initiation and progression [4,40]. Here, we demonstrated that the overall OCR and ECAR were considerably repressed by miR125/SLN-KL, E, E/LPN, and E/LPN-KL treatments to varying degrees compared with those found in the control group (Figure 3d,e). This result suggested the inhibitory effect of these formulations on aerobic glycolysis in SAS cells. The most remarkable reduction was observed in the miR125/SLN-KL+E/LPN-KL group (Figure 3d,e).

### 3.4. Increase in Glucose Uptake in SAS Cells Treated with Different Nanoparticle Formulations

**Figure 4:**
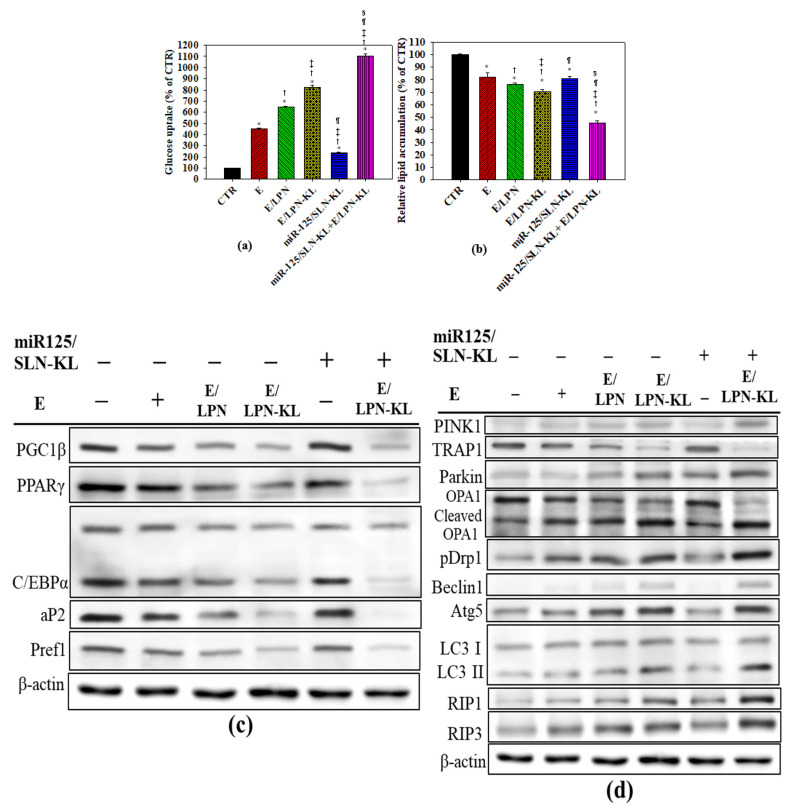
Effect of different formulations on the relative levels of (**a**) glucose uptake and (**b**) lipid accumulation. For (**a**,**b**): * *p* < 0.05: compared with CTR, ^†^ *p* < 0.05 compared with E/LPN, ^‡^ *p* < 0.05 compared with E/LPN, ^¶^ *p* < 0.05 compared with E/LPN-KL, and ^§^ *p* < 0.05 compared with miR125/LPN-KL via Student’s *t*-test analysis, respectively. (**c**,**d**) The expression of proteins associated with (**c**) lipid or glucose metabolism and (**d**) mitochondrial dynamics, fusion/fission, mitophagy, and necroptosis in SAS cells. After various treatments, the cells were evaluated with Western blot, and the representative blots were shown (*n* = 3).

When SAS cells were treated with E, E/LPN, E/LPN-KL, miR-125/SLN-KL, and E/LPN-KL+miR-125/SLN-KL, the glucose uptake into cells was significantly elevated compared to the control group in SAS cells (Figure 4a). Furthermore, when SAS cells were treated with E/LPN-KL+miR-125/SLN-KL, the cells showed the most significant increase in the glucose uptake compared to the control group (Figure 4a).

### 3.5. Decreased Accumulation of Oil Droplets in SAS Cells Treated with Different Nanoparticle Formulations

When the cells were treated with E and miR-125-loaded formulations, the accumulation of oil droplets in the cells significantly decreased compared with that in the control group, especially in the treatment group of E/LPN-KL (Figure 4b). Furthermore, the cells co-treated with E/LPN-KL and miR-125/SLN-KL exhibited the most remarkable reduction in the lipid droplet accumulation among all the groups (Figure 4b).

### 3.6. Evaluation of Proteins Associated with Adipogenesis and Lipid Synthesis by Western Blot

Figure 4c illustrates that E-containing formulations downregulated adipogenic factors, such as preadipocyte factor 1 (Pref1), peroxisome proliferator-activated receptor-gamma (PPARγ), PPARγ coactivator 1α (PGC1α), the transcription factor cAMP response element-binding protein α (C/EBPα), and adipocyte protein 2 (aP2), to varying degrees at the protein level. Strikingly, the combined treatment of E/LPN-KL and miR125/SLN-KL most significantly suppressed the expression of these proteins associated with adipogenesis and lipid synthesis among all the treatment groups (Figure 4c). E-loaded formulations, particularly E/LPN-KL and its co-treatment with miR125/SLN-KL, inhibited the expression of PGC1ß. Thus, thermogenesis might be suppressed in SAS cells (Figure 4c).

### 3.7. Assessment of Proteins Related to Mitophagy and Necropotosis by Western Blot

Interestingly, when SAS cells were treated with E- and miR125-loaded formulations, mitochondrial autophagy/mitophagy-related proteins were altered to various extents. The expression of PINK1/Parkin substantially increased, but the expression of tumor necrosis factor receptor associated protein 1 (TRAP1) decreased in response to the treatment of E and miR125, especially for the co-treatment of E/LPN-KL and miR125/SLN-KL (Figure 4d). Optic atrophy 1 (OPA1), a mitochondrial dynamin-like GTPase, was cleaved, whereas the phosphorylation of dynamin related protein (DRP) 1, a mitochondrial fission marker, was increased in response to the treatment of E/LPN-KL and/or miR125/SLN-KL (Figure 4d). Moreover, autophagy- and necropotosis-related proteins, including microtubule-associated protein 1 light chain 3 (LC3) II, Beclin 1, autophagy related 5 (Atg5), and RIP1/3, were upregulated after incubation with E and miR125, particularly for the combination therapy of E/LPN-KL and miR125/SLN-KL.

### 3.8. Reduced Migration of SAS Cells Treated with E- and miR-125-Loaded Formulations

**Figure 5:**
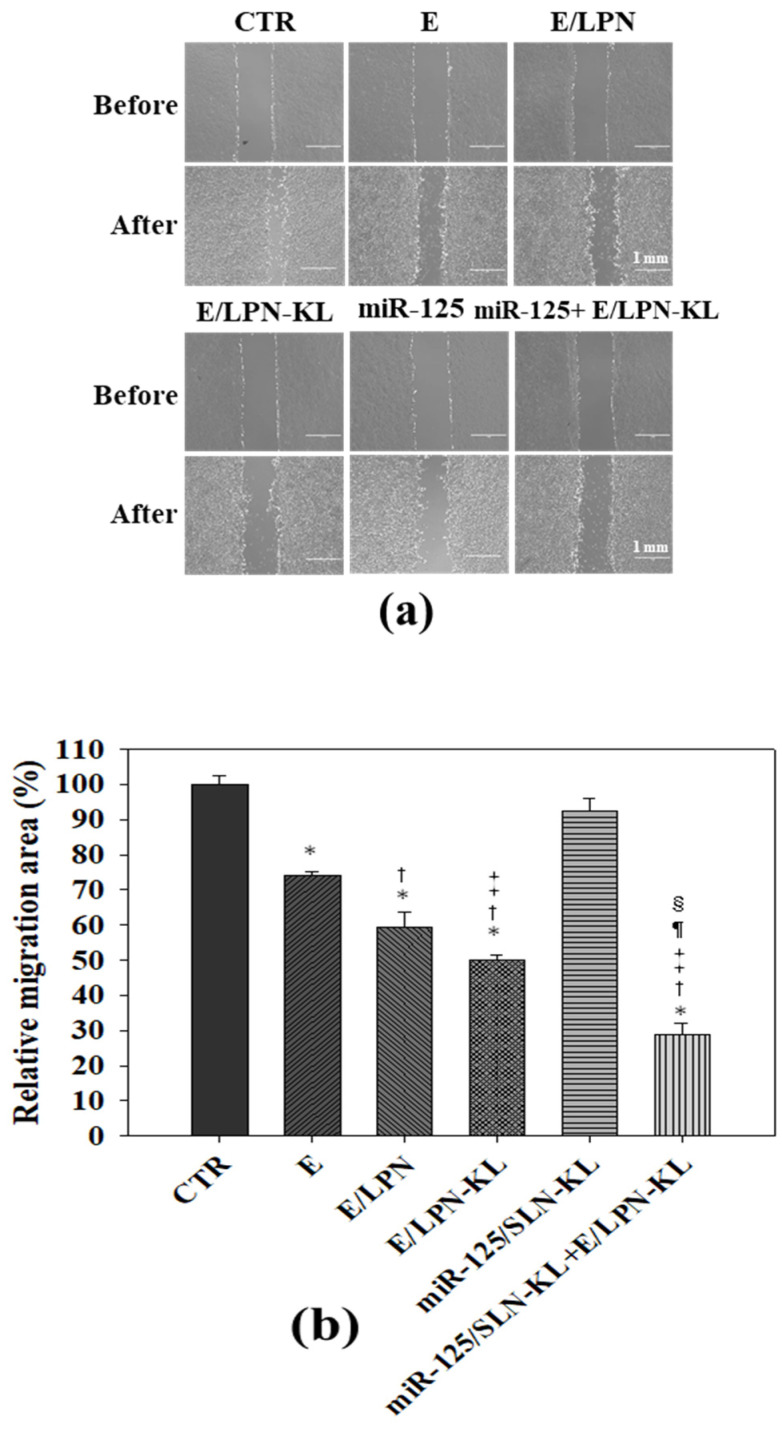

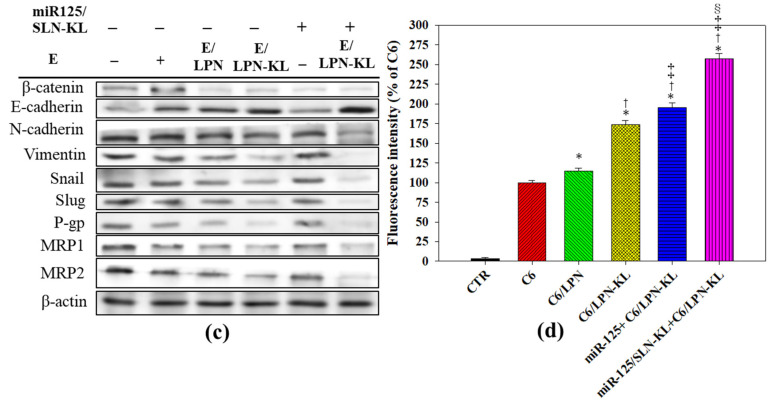
Effect of various treatments on migration and multidrug resistance (MDR) pathway in SAS cells**.** (**a**) The migration assay was conducted to evaluate the SAS cells treated with different formulations of E and miR-125 for 15 h. Cellular images were then observed under a microscope. (**b**) Relative percentages of the cell migration area. * *p* < 0.05: compared with CTR, ^†^ *p* < 0.05 compared with E/LPN, ^‡^ *p* < 0.05 compared with E/LPN, ^¶^ *p* < 0.05 compared with E/LPN-KL, and ^§^ *p* < 0.05 compared with miR125/LPN-KL via Student’s *t*-test analysis. (**c**) The protein expression levels of epithelial–mesenchymal transition (EMT) and MDR were evaluated via Western blot after the SAS cells were given various treatments for 48 h. The experiments were performed in triplicate with similar results. (**d**) The cellular uptake of coumarin-6 (C6; a probe of E) was monitored by detecting the relative green fluorescence intensity of C6 by using a flow cytometer. * *p* < 0.05 compared with C6, ^†^ *p* < 0.05 compared with C6/LPN, ^‡^ *p* < 0.05 compared with C6/LPN-KL, and ^§^ *p* < 0.05 compared with miR125+C6/LPN-KL via Student’s *t*-test analysis, respectively.

The result of the migration assay performed on Transwell inserts showed that the treatment with free E mildly decreased cell migration by approximately 30% (Figure 5a,b). The migration area of SAS cells was further reduced by the treatment of E/LPN or E/LPN-KL (Figure 5a,b). Notably, the combined treatment of miR125/SLN-KL and E/LPN-KL further diminished the cell migration percentage to 28.94% ± 2.96% (Figure 5a,b). The protein expression levels of *β*-catenin, EMT, and multidrug resistance (MDR) pathways in SAS cells were assessed via Western blot after these cells were treated with miR-125- and E-incorporated preparations. Lone miR-125- and E-loaded formulations attenuated the protein expression associated with *β*-catenin, EMT, and MDR pathways to varying degrees. Moreover, the co-modulation by miR125/SLN-KL and E/LPN-KL remarkably abolished the expression of *β*-catenin, N-cadherin, vimentin, Snail, Slug, P-glycoprotein (P-gp), MDR-associated protein (MRP)1, and MRP2 and markedly intensified the E-cadherin expression compared with those in the other treatment groups (Figure 5c). These results indicated the superior suppressive effect of the co-treatment of miR125/SLN-KL and E/LPN-KL on the metastasis and resistance of SAS cells.

We further evaluated the possible cellular uptake enhancement of E in different formulations into SAS cells. The intracellular intensity of C6, a green fluorescent marker of E, after being transported by various LPN formulations, was measured using a flow cytometer. In Figure 5d, a significant difference was observed when C6 was delivered by LPN or LPN-KL (*p* < 0.05). After the combined treatment with miR125/SLN-KL, the cellular intensity of C6/LPN-KL remarkably increased compared with that of the group without co-treatment. Interestingly, the group of miR125/SLN-KL+C6/LPN-KL exhibited the highest fluorescence intensity of C6 among the groups (*p* < 0.05; Figure 5d).

### 3.9. Cytotoxicity of E in Various Formulations on NOK and SAS Cells

**Figure 6:**
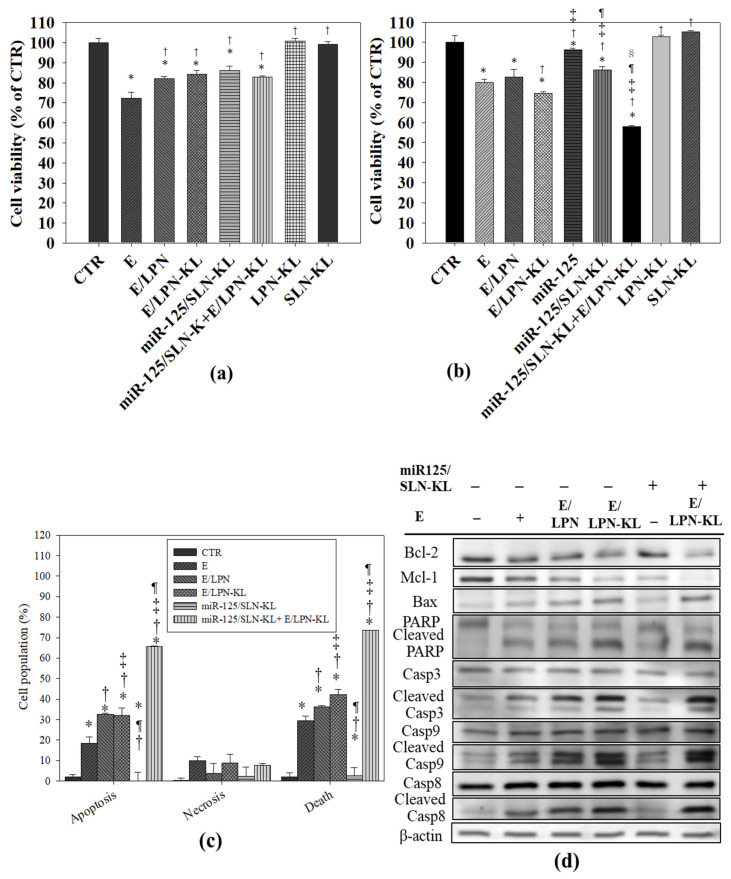
(**a**,**b**) Cytotoxicity of E and miR-125 in various formulations on normal and cancer cells. (**a**) NOK (normal oral keratinocyte) and (**b**) SAS cells were treated with E and/or miR-125 in various formulations for 48 h. Cell viability was measured with sulforhodamine B (SRB) assay. Values are the mean ± SD (*n* = 3). For (**a**): * *p* < 0.05 compared with CTR; ^†^ *p* < 0.05 compared with E. For (**b**): * *p* < 0.05 compared with CTR; ^†^ *p* < 0.05 compared with E; ^‡^ *p* < 0.05 compared with E/LPN-KL; ^¶^ *p* < 0.05 compared with miR-125; ^§^ *p* < 0.05 compared with miR-125/SLN-KL. (**c**) The relative percentage of the apoptotic, necrotic, and dead cell population by Annexin V and propidium iodide (PI) assay. * *p* < 0.05 compared with CTR; ^†^ *p* < 0.05 compared with E; ^‡^ *p* < 0.05 compared with E/LPN; ^¶^ *p* < 0.05 compared with E/LPN-KL. (**d**) Effect of different treatments on the expression of proteins associated with the apoptosis of SAS cells. After various treatments, the cells were evaluated with Western blot, and their representative images were shown (*n* = 3).

NOK cells were used to evaluate the possible toxic effects of these miR-125- and E-loaded formulations on normal cells by using the SRB assay. We selected the E concentration of 20 μM, which was approximately IC30 (the concentration of 30% inhibition of cell viability) of E on NOK cells and IC20 of E on SAS cells (Figure 6a,b). E was encapsulated in various formulations in the presence and absence of miR125/SLN-KL, and their cytotoxic effects on NOK cells after 48 h of incubation were compared with those of the control and E groups (Figure 6a). The result showed that the cell viability after the treatment with E/LPN or E/LPN-KL with or without miR125/SLN-KL increased to approximately 80–85% (Figure 6a). Therefore, E/LPN-KL or its co-treatment with miR125/SLN-KL reduced the toxic effects of free E on NOK cells (Figure 6a).

We tested the effect of E and/or miR-125-loaded formulations on the viability of SAS cells. We found that 20 μM E diminished the cell viability of SAS cells to 79.97%. E/LPN-KL decreased the viability of SAS cells to 74.70% (relative percentage of the control group; Figure 6b). Interestingly, E/LPN-KL+miR-125/SLN-KL further reduced the viability of SAS cells to 57.88% ± 0.67% (Figure 6b). Notably, E/LPN-KL+miR-125/SLN-KL displayed the highest inhibitory effect on SAS cell viability among all these formulations (Figure 6b).

### 3.10. Apoptotic Effect of E- and miR-125-Loaded Formulations on SAS Cells

Cell apoptosis and necrosis (%) were assessed via Annexin V—PI double staining on SAS cells. The result indicated that the apoptosis percentage (% of the sum of early and late apoptotic cells) and death percentage (% of the sum of apoptotic and necrotic cells; Figure 6c) activated by E/LPN-KL were substantially higher than those triggered by E (*p* < 0.05). Noticeably, the number of apoptotic and dead cells due to the combined treatment of E/LPN-KL and miR125/SLN-KL was considerably more than that of the cells subjected to the other treatments (Figure 6c). Furthermore, marginal differences in the low percentage of the necrotic population were observed among all the formulations (*p* > 0.05; Figure 6c).

The protein expression of the apoptosis-associated pathway of SAS cells after 48 h of treatments was assessed via Western blot. Myeloid cell leukemia 1 (Mcl-1) and Bcl-2, two anti-apoptotic proteins, were marginally inhibited by E but were significantly suppressed by the combined treatment of E/LPN-KL and miR125/SLN-KL (Figure 6d). On the contrary, the expression levels of pro-apoptotic proteins, such as Bax, cleaved poly (ADP-ribose) polymerase (PARP), caspase-3, caspase-8, and caspase-9, were slightly increased by the lone treatment of E, but these apoptosis-inducing proteins were progressively increased by the treatments of E/LPN-KL and miR125/SLN-KL (Figure 6d).

### 3.11. Serum Cholesterol and Glucose Levels In Vivo

**Figure 7:**
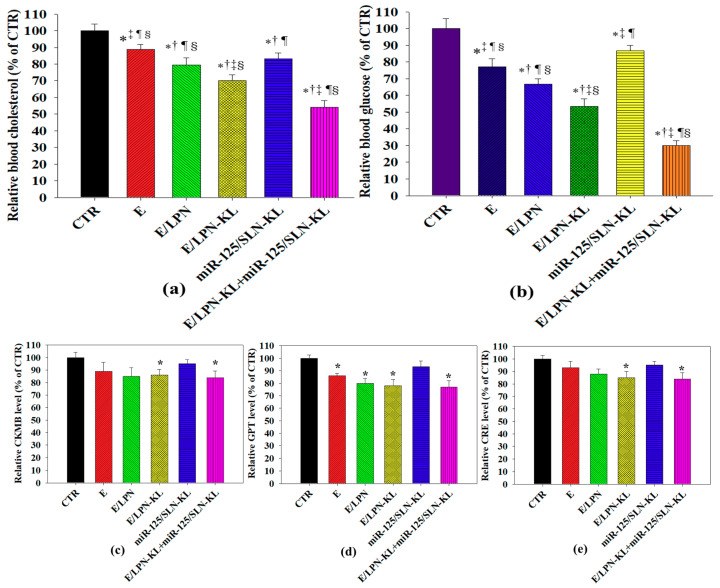
In vivo quantitation of blood (**a**) cholesterol, (**b**) glucose, (**c**) creatine kinase-myocardial band (CKMB), (**d**) glutamate pyruvate transaminase (GPT), and (**e**) creatinine (CRE) levels in SAS-bearing mice in different treatment groups. * *p* < 0.05 compared with CTR, ^†^ *p* < 0.05 compared with E, ^‡^ *p* < 0.05 compared with E/LPN, ^¶^ *p* < 0.05 compared with E/LPN-KL, and ^§^ *p* < 0.05 compared with miR-125/SLN-KL.

On the basis of our in vitro data, we further investigated the effect of E, E/LPN, E/LPN-KL, miR-125/SLN-KL, and E/LPN-KL+miR-125/SLN-KL formulations on blood cholesterol and glucose levels in mice (Figure 7a,b). When SAS-bearing mice were treated with various formulations, the cholesterol and glucose levels in all the treatment groups of E, E/LPN, E/LPN-KL, miR-125/SLN-KL, and E/LPN-KL+miR-125/SLN-KL were significantly reduced compared with those in the control group (Figure 7a,b). The most significant decrease in serum cholesterol and glucose levels was found in the combined group of E/LPN-KL and miR-125/SLN-KL (Figure 7a,b).

### 3.12. In Vivo Biosafety Evaluation

Serum CKMB, GPT, and CRE levels were monitored to examine the heart, liver, and renal functions, respectively (Figure 7c–e). Marginal or mild changes were observed in these three serum parameters after the treatment with E, E/LPN, or E/LPN-KL or its co-treatment with miR125/SLN-KL. Nevertheless, no substantial alterations were detected in these three parameters (Figure 7c–e). Interestingly, E has elicited a hepatoprotective effect against valproic acid-provoked hepatic injury in rats [41]. Our formulations did not cause serious organ toxicities and thus did not have obvious effects to alleviate the heart-, liver-, and kidney-related toxicities.

### 3.13. In Vivo Antitumor Efficacy and Body Weight Studies on SAS Tumor-Bearing Mice

**Figure 8:**
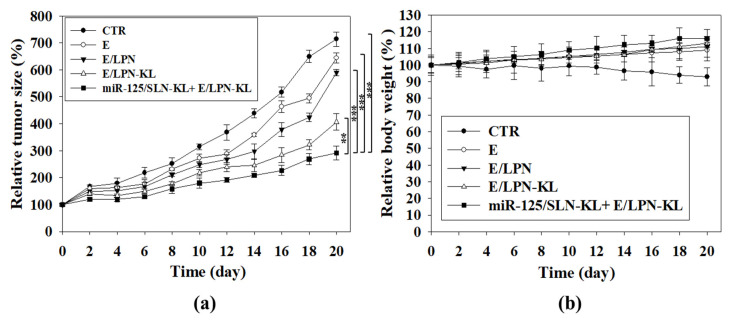
Antitumor efficacy and body weight studies on SAS-bearing mice treated with different formulations. (**a**) Antitumor efficacy of various formulations intravenously injected into SAS-bearing mice. Tumor growth was measured with digital calipers every 2 days (statistical significance: ** *p* < 0.01; *** *p* < 0.001.); (**b**) body weight as a function of time in CT-26-bearing mice.

The antitumor efficacy of various E- and miR-125-loaded formulations was detected in SAS tumor-bearing mice in vivo. The combined treatment of tumor-targeted E/LPN-KL and miR125/SLN-KL demonstrated the most noteworthy antitumor efficacy on SAS-bearing mice. This result suggested the greatest tumor-suppression ability of these combinatorial nanoparticle formulations among the different treatment groups (Figure 8a). For the safety evaluation, the body weight of SAS-bearing mice was also assessed. The untreated mice showed a mild decrease in body weight, possibly because of the increasing tumor burden (Figure 8b). All the other groups exhibited a constant increase in body weight and slight individual differences (Figure 8b), indicating the improvement in tumor burden-related weight loss.

## 4. Discussion

The mitochondria of cancer cells usually have altered physiological processes, including hindered oxidative phosphorylation and mitochondrial hyperpolarization [4]. Furthermore, cancer cells demonstrate the atypical metabolism of the Warburg effect by displaying higher glycolysis and lactate production levels and misbalanced mitochondrial ATP generation [42]. These changes may affect the regulation of bioenergetics and the cellular oxidation-reduction (redox) equilibrium [3], leading to tumorigenesis, progression, angiogenesis, and chemotherapy resistance in cancer cells [43]. However, information about the regulation of these complicated factors of the mitochondrial bioenergetics on modulating HNC cell death is limited. Hence, in the present study, we aimed to evaluate the modulating effects of E/LPN-KL and miR125/SLN-KL on mitochondrial dynamics, energy metabolism, and anticancer efficacy in SAS cells.

E is abundant in oak, nuts, and fruits [14]. E in chestnut extract inhibits cell proliferation, induces apoptosis, modulates mitochondrial depolarization, and affects the cytokinomic and metabolomic properties of HepG2 cells [44]. Besides, miR-125 triggers pluripotent regulation in a wide variety of cancers, such as CRC, HNC, and hepatocellular carcinoma (HPC) [18,19,20]. Accordingly, the miR-125 mimic induces CRC cell apoptosis and reduces cell viability [19]. Furthermore, miR-125 upregulation in OSCC causes escalated oxidative stress and improves drug sensitivity against OSCC [45]. In particular, miR-125 transfection re-sensitizes HPC cells to 5-FU, possibly through the inhibition of glucose uptake and lactate production by directly targeting hexokinase II [46].

In this study, we used MitoTracker to verify the targeting delivery of E and miR-125 to the mitochondria via the formulation of LPN-KL or SLN-KL, respectively (Figure 2a,b). We found that the increased ROS generation (Figure 3a) and mitochondrial depolarization (Figure 3b) caused mitochondrial dysfunction in SAS cells (Figure 3c–e). The formulation of miR125/SLN-KL+E/LPN-KL most significantly disrupted mitochondrial respiration, aerobic glycolysis, and cellular bioenergetics, including OCR, ECAR, and ATP production (Figure 3c–e), indicating the excellent efficacy of this combinatorial therapy on modulating mitochondrial bioenergetics and dynamics in SAS cells.

Lipid production and accumulation are important for tumor progression because these processes may produce energy for tumorigenesis and provide the building components of phospholipids to form cell membranes [47]. PGC-1, a transcriptional coactivator, is the major regulator of mitochondrial bioenergenesis associated with energy metabolism by interacting with the nuclear receptor PPAR-*γ* and C/EBPα [48,49]. PGC-1*β* is pivotal to activate fatty acid *β*-oxidation [47]. Figure 4 shows that the co-treatment of E/LPN-KL and miR125/SLN-KL remarkably increased glucose uptake (Figure 4a) and significantly reduced lipid accumulation (Figure 4b) in SAS cells. The combinatorial therapy also considerably diminished the protein expression levels of adipogenic factors and metabolism of lipids, including Pref1, PGC1ß, PPARγ, C/EBPα, and aP2, among all the treatment groups (Figure 4c). This result implied that this combined formulation might disturb lipid metabolism and mitochondrial bioenergenesis, thereby suppressing tumor progression. Figure 4c is consistent with the result of Woo et al. [50], who suggested that E might regulate adipogenesis at the early and middle stages to inhibit lipid synthesis [50]. Moreover, pomegranate E modulates lipid accumulation and cholesterol metabolism in human hepatic cells by regulating the PPAR*γ*-related pathway [51]. Additionally, the hepatic removal of PGC-1*β* modulates anabolic metabolism, thereby potentiating ROS-provoked damages of DNA, proteins, and lipids [52]. Consequently, the mitochondrial dynamic alteration induces cancer cell apoptosis and inhibits HPC tumor growth in mice [52].

The balance between two important housekeepers, namely, Parkin-mediated mitophagy and fusion/fission, is critical to maintaining mitochondrial homeostasis [4]. Mitophagy is provoked by numerous stimuli, such as oxidative stress and increased oxidative phosphorylation activities [4]. Mitophagy can be regulated by different upstream regulators, such as PINK1, TRAP1, and Parkin, to induce cancer cell death [4]. Furthermore, treatment with matrine, a natural alkaloid, modulates the PINK1/Parkin pathways and altered protective mitophagy, thereby provoking mitochondrial apoptosis in HPC HepG2 cells [53]. Our result indicated that PINK1/Parkin was upregulated, whereas TRAP1 was downregulated in response to the treatment of E/LPN-KL and miR125/SLN-KL (Figure 4d). This result revealed that the co-treatment of miR-125 and E in mitochondrion-targeted nanoparticles might regulate mitophagy by modulating the pathways of mitochondrial remodeling (i.e., PINK1/Parkin) and oxidative stress (i.e., TRAP1) in SAS cells. Furthermore, we found OPA1, a mitochondrial fusion marker, was cleaved, but p-Drp1, a mitochondrial fission marker, increased after the treatment of E/LPN-KL and miR125/SLN-KL (Figure 4d). This result suggested that mitochondrial fission was more favorable than fusion to the induction of apoptosis (Figure 6c,d). Moreover, this combination therapy increased the protein levels of LCII, Beclin 1, Atg5, and RIP1/3, indicating that the co-treatment of miR-125 and E in nanoparticle preparations might regulate mitochondrial autophagy/mitophagy and necropotosis pathways in SAS cells.

Intriguingly, another study has shown that Krüppel-like factor 4, a zinc finger transcription factor, considerably increases ROS levels and triggers mitochondrial fusion, thus inducing the G2/M cell cycle arrest and protecting cells from nutrient deprivation-induced death in human glioblastoma cells [54]. Consistent with our current finding, previous evidence has suggested that excessive mitochondrial fission may happen during cell metabolism, proliferation, apoptosis, and migration through the activation of the intrinsic (mitochondrial) apoptotic pathway [3]. Accordingly, the Drp1 post-transcriptional phosphorylation and alteration of the Parkin transcription activity via p53 may trigger mitochondrial fission and aberrant mitophagy [4]. Such mitophagy defect may provoke cellular oxidative stress, energy metabolism imbalance, and calcium overload; consequently, it induces caspase 9-mediated intrinsic (mitochondrial) apoptosis and abolishes F-actin-dependent cellular migration in cancer cells [6]. Correspondingly, our result implied that the treatment of E/LPN-KL and miR125/SLN-KL considerably suppressed SAS cell migration and MDR by inhibiting *β*-catenin, N-cadherin, vimentin, snail, and Slug in the EMT pathway and P-gp and MRP1/2 in the MDR pathway (Figure 5). The mitochondria are responsible for the dynamic equilibrium of various pro-apoptotic and anti-apoptotic proteins, such as Bcl-2 and Bax [43]. Remarkably, the co-treatment of E/LPN-KL and miR125/SLN-KL significantly induced the SAS cell apoptosis by effectively inhibiting Bcl-2 and Mcl-1 and by inducing the cascade cleavage of PARP, caspase-3, caspase-8, and caspase-9 (Figure 6). These results confirmed the potential of E/LPN-KL and miR125/SLN-KL for application in overcoming migration and MDR while enhancing apoptosis and increasing cytotoxicity against SAS cells (Figure 5 and Figure 6). The safety evaluation in NOK cells also suggested the protective effect elicited by LPN-KL nanovehicles to decrease the toxic effects of E on noncancerous cells (Figure 6b).

Encouraging preclinical findings suggested that the co-treatment of E/LPN-KL and miR125/SLN-KL given to SAS-bearing mice reduced blood glucose and cholesterol levels (Figure 7a,b), indicating that E/LPN-KL and miR125/SLN-KL might modulate the pathways associated with tumor lipid and glucose metabolism and, consequently, disturb tumor progression and growth. Concomitantly, the co-treatment of E/LPN-KL and miR125/SLN-KL reduced the tumor size in SAS-bearing mice compared with that of CTR, E, E/LPN, and E/LPN-KL (*p* < 0.001–0.01; Figure 8a,b). These results validated that targeting EGFR of SAS-tumor cells and effective intracellular and mitochondrial delivery are key prerequisites for lipid nanoparticles, such as LPN-KL and SLN-KL, to display a superior antitumor efficacy and excellent hypolipidemic and hypoglycemic effects in vivo. No obvious changes in the body weight and biochemical markers of the heart, liver, and kidney were observed in the treatment groups (Figure 7c–e and Figure 8b). These results suggested the acceptable biosafety of the E- and miR-125-containing formulations. This finding was also consistent with the subchronic toxicity evaluation of E as a food additive in F344 rats; that is, no death or treatment-associated clinical symptoms were detected during the experimental period [55].

## 5. Conclusions

We developed EGFR-targeting and mitochondrion-directed nanoparticles to incorporate E and miR-125 for the modulation of lipid and glucose metabolism, mitochondrial oxidative stress, remodeling, bioenergenesis, mitophagy, and fusion/fission dynamics. On the basis of the data about mitochondrial respiration and aerobic glycolysis, we demonstrated that the inhibitory effect of miR125/SLN-KL and E/LPN-KL on cellular bioenergetics was highly correlated with their anticancer activity. These formulations also regulated multiple pathways of tumor metabolism, mitochondrial dynamics, apoptosis, resistance, and metastasis to promote the sensitivity of SAS cells to E and miR-125. These dosage forms also elicited low toxic effects on normal cells. To our best knowledge, this prospective study can be used as a basis for combining miR-125 with natural compounds, such as E, in nanoformulations to regulate mitochondrial dynamics and energy metabolism associated with cancer.

## Figures and Tables

**Table 1:** Characteristics of E/LPN-KL and miR-125/SLN-KL.

Formulation	Particle Size (nm)	PdI^a^	Zeta Potential (mV)	EE^b^ (%)
E/LPN-KL	195.23 ± 5.88	0.24 ± 0.08	−18.73 ± 2.29	85.53 ± 1.35
miR-125/SLN-KL	158.67 ± 3.69	0.20 ± 0.06	46.47 ± 1.22	86.28 ± 1.56

PdI^a^: polydispersity index; EE^b^: encapsulation efficiency.

## Supplementary Materials

The following are available online at https://www.mdpi.com/1999-4923/12/8/756/s1, Table S1: Antibodies used in this study.
