# Supplementary material for: Mitochondrion-Directed Nanoparticles Loaded with a Natural Compound and a microRNA for Promoting Cancer Cell Death via the Modulation of Tumor Metabolism and Mitochondrial Dynamics
Source: Pharmaceutics. 2020 Aug 11;12(8):756. doi: 10.3390/pharmaceutics12080756 (PMC7464316; doi:10.3390/pharmaceutics12080756)
Supplement: Supplementary file 1 [file pharmaceutics-12-00756-s001.pdf]

pharmaceutics

Supplementary Materials: Mitochondrion-Directed Nanoparticles Loaded with a Natural Compound and a microRNA for Promoting Cancer Cell Death via the Modulation of Tumor Metabolism and Mitochondrial Dynamics

Yu-Li Lo *, Chen-Shen Wang, Yen-Chun Chen, Tse-Yuan Wang, Yih-Hsin Chang, Chun-Jung Chen and Ching-Ping Yang

Table S1. Antibodies used in this study.

Antibody	Company	Catalog
Anti-Bax	GeneTex	GTX109683
Anti-Bcl-2	GeneTex	GTX127958
Anti-PARP	Cell Signaling	9542
Anti-Caspase 3	Cell Signaling	9662
Anti-Snail	Cell Signaling	C15D3
Anti-Slug	Abcam	Ab75629-100
Anti-E-cadherin	Cell Signaling	3195
Anti-β-catenin	ProteinTech	51067-2-AP
Anti-P-gp	GeneTex	GTX108370
Anti-MRP1	GeneTex	GTX116046
Anti-β-actin	Millipore	2640549
Anti-C-EBPα	Cell Signaling	8178
Anti-PPARγ	Cell Signaling	C26H12
Anti-Pref-1	Cell Signaling	2069
Anti-PINK1	Bio Vision	3929-30T
Anti-TRAP1	GeneTex	GTX102017
Anti-Atg5	Cell Signaling	12994
Anti-Beclin 1	Sino	105332-T40
Anti-RIP1	GeneTex	GTX111074
Anti-Parkin	GeneTex	GTX65811
Anti-OPA1	Cell Signaling	80471
Peroxidase-conjugated AffiniPure Goat Anti-Rabbit IgG	Jackson	AB_2307391
